# Nuclear re-localization of Dicer in primary mouse embryonic fibroblast nuclei following DNA damage

**DOI:** 10.1371/journal.pgen.1007151

**Published:** 2018-02-02

**Authors:** Kaspar Burger, Monika Gullerova

**Affiliations:** Sir William Dunn School of Pathology, University of Oxford, Oxford, United Kingdom; University of Cambridge, UNITED KINGDOM

## Abstract

Dicer is a key component of RNA interference (RNAi) and well-known for its role in biogenesis of micro (mi)RNA in the cytoplasm. Increasing evidence suggests that mammalian Dicer is also present and active in the nucleus. We have previously shown that phosphorylated human Dicer associates with chromatin in response to DNA damage and processes double-stranded (ds)RNA in the nucleus. However, a recent study by Much et al. investigated endogenously tagged HA-Dicer both in primary mouse embryonic fibroblasts (PMEFs) as well as adult homozygous viable and fertile HA-Dicer mice under physiological conditions and concluded that murine Dicer is exclusively cytoplasmic. The authors challenged several findings, reporting functions of Dicer in mammalian nuclei. We have re-investigated this issue by applying subcellular fractionation, super-resolution microscopy followed by 3D reconstitution, and phospho-Dicer-specific antibodies using the same HA-Dicer PMEF cell line. Our data show that a small fraction of the murine HA-Dicer pool, approximately 5%, localises in the nucleus and is phosphorylated upon DNA damage. We propose that Dicer localisation is dynamic and not exclusively cytoplasmic, particularly in cells exposed to DNA damage.

## Author summary

Cytoplasmic Dicer is a key component of the canonical micro (mi)RNA biogenesis pathway. However, a growing body of evidence points toward localisation and activity of mammalian Dicer in the nucleus. A recent study by Much et al., employed an endogenously HA-tagged Dicer knock-in mouse cell line to show that Dicer is exclusively cytoplasmic. This paper challenges several studies reporting various RNA metabolic functions of Dicer in human nuclei. Given the controversy about Dicer’s subcellular localisation, it is essential to address this issue. Employing the same cells as used by Much and colleagues, we combined super-resolution microscopy followed by 3D reconstitution and biochemical assays to show that endogenously tagged HA-Dicer prominently localises in the nucleus under physiological conditions. We demonstrate that DNA damage triggers accumulation of phosphorylated HA-Dicer in the nucleus, confirming previous observations in human cells. Our data indicate evolutionary conservation of nuclear Dicer localisation and function in mammals in response to DNA damage.

## Introduction

The endoribonuclease Dicer recognises and processes double-stranded (ds)RNA substrates of various origins into small non-coding (nc)RNA [1]. Dicer activity generates 20–25 nt long micro (mi)RNA from precursors and modulates gene expression by post-transcriptional gene silencing (PTGS) in the cytoplasm (reviewed in [2]). A growing body of evidence suggests that additional functions for Dicer may exist in many species, including mammals, which are potentially independent of miRNA biogenesis and may involve non-canonical modes of RNAi in the nucleus (reviewed in[3]). Conditional depletion of Dicer in mouse embryonic stem cells, for instance, compromises centromere silencing and impairs expression of homologous endogenous dsRNA loci [4, 5]. A series of studies imply nuclear localisation of mammalian Dicer and association with chromatin. The Filipowicz lab reported enrichment of mammalian Dicer at ribosomal RNA loci, suggesting a possible role for Dicer in maintaining integrity of ribosomal DNA arrays. However, the authors could not describe a direct function for Dicer in nucleoli [6]. Human Dicer may also interact with the nuclear pore complex component NUP153 [7]. Interestingly, Dicer depletion in human cells caused defects in precursor messenger (pre-m)RNA processing [8]. Catalytically active Dicer was purified from human nuclei and shown to promote processing of dsRNA hairpin structures [9] and stimulate initiation of RNAPII transcription at hormone-responsive genes [10]. In addition, nuclear Dicer fosters termination of RNAPII transcription [11] and alternative polyadenylation at a subset of protein-coding genes [12]. The latter two studies conclude that Dicer association with chromatin may be mediated by the localised production of dsRNA, which is processed into endogenous small interfering (endo-si)RNA to mediate heterochromatin formation by recruitment of G9a methyltransferase in a Dicer-dependent manner. These findings are in line with previous studies, reporting the existence of nuclear RNAi in human cells [13]. The authors showed that transfection of exogenous small interfering (exo-si)RNA triggers silencing of a subset of protein-coding gene promoters. More recently, two studies point toward Dicer-dependent nuclear RNAi in mammals by demonstrating that nuclear, chromatin-associated Dicer impairs expression of the microtubule-binding protein Doublecortin in mouse adult neural stem cells [14] and transactivation of the human secreted frizzled-related protein 1 promoter in cholangiocarcinoma cells [15]. Collectively, these data indicate that Dicer may be present and active in mammalian nuclei to regulate expression of protein-coding genes by both miRNA-dependent and -independent mechanisms.

However, mechanistic insight in mammalian nuclear Dicer localisation remains largely inconclusive. Analysis of ectopically expressed human Dicer mutants suggest that the dsRNA binding domain (dsRBD) may harbour a cryptic nuclear localisation signal, which is potentially occluded by the helicase domain in the full-length Dicer protein [16]. Indeed, lack of the helicase domain or duplication of the dsRBD trigger nuclear accumulation of ectopically expressed Dicer mutants [16]. Confusingly, the authors could not detect nuclear localisation of full length Dicer under physiological conditions. The N-terminal Dicer helicase domain forms a clamp-like structure adjacent to the RNase III active site in the base of the Dicer enzyme [17]. Truncation of the helicase domain or alterations of C-terminal domains, such as introduction of post-translational modifications, may cause structural rearrangements that ‘unfold’ the helicase domain, potentially exposing an ‘unmasked’ C-terminal domain for increased dsRNA binding affinity and catalytic activity. However, recent data demonstrate that a different cytoplasmic N-terminal deletion mutant of human Dicer efficiently processes exogenous dsRNA substrates in HEK293-derived Dicer knockout cells, but fails to accumulate to the nucleus [18], indicating that rearrangement of the Dicer helicase domain is necessary, but not sufficient for nuclear accumulation.

Moreover, recent work by Much et al. challenged the existence of mammalian Dicer in the nucleus *per se* [19]. Using PMEF::HA-Dicer cells, a primary mouse embryonic fibroblast cell line, which expresses a catalytically active, endogenously HA-tagged Dicer (HA-Dicer) at physiological levels [20], the authors failed to detect any evidence for nuclear HA-Dicer localisation under conditions that were previously reported to trigger nuclear Dicer accumulation, such as treatment with the nuclear export inhibitor Leptomycin B (LMB), stimulation of mitogen-activated protein kinase (MAPK) signalling or DNA damage-inducing γ-irradiation. These findings seem to contradict various other subcellular localisation studies, which apparently detect a fraction of Dicer in the nucleus of human cells [21, 22] and in purified nuclei [23]. In this regard, we have recently shown that a subset of the endogenous human Dicer pool is phosphorylated in response to DNA damage and associates with DNA double-strand breaks (DSBs) on chromatin to process damage-induced dsRNA [24]. Similarly, human Dicer may be recruited to DNA lesions to mediate chromatin decondensation during nucleotide excision repair in response to UV irradiation [25]. These findings suggest a functional link between nuclear Dicer accumulation and the DNA damage response (DDR).

Here, we provide evidence for existence of HA-Dicer in murine nuclei under physiological conditions and involvement of nuclear phosphorylated HA-Dicer in the DDR. Using subcellular fractionation, super-resolution microscopy followed by 3D reconstitution and phospho-Dicer-specific antibodies, we demonstrate that a small fraction of HA-Dicer localises to nuclei of unperturbed cells. Following DNA damage, phosphorylated HA-Dicer accumulates in the nucleus in a phosphatidylinositol-3-kinase (PI3K)-dependent manner. We propose that a subset of the mammalian Dicer pool relocalises to the nucleus rather than being exclusively restricted to the cytoplasm.

## Results and discussion

**Fig 1:**
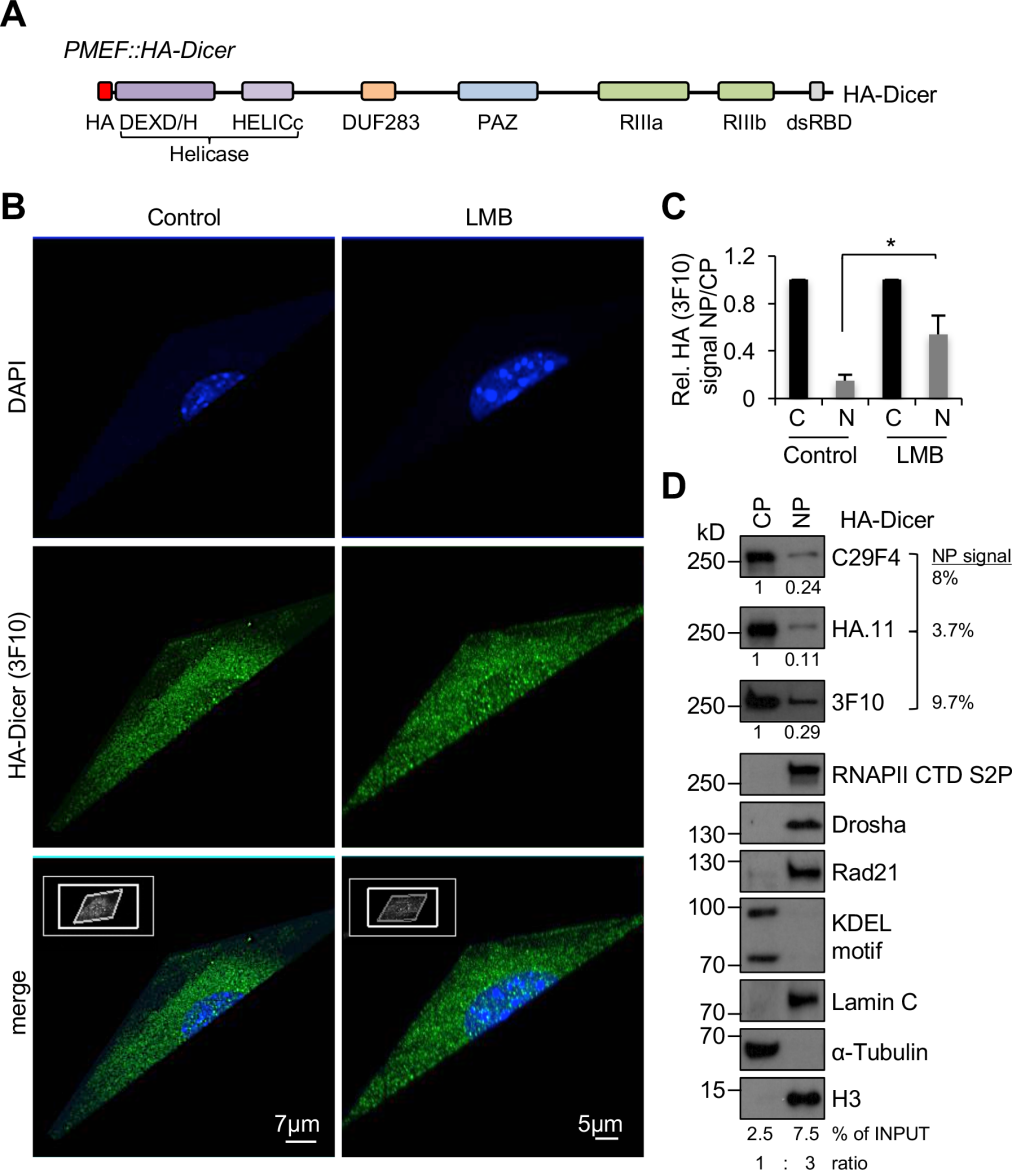
Subcellular localisation of HA-Dicer in murine PMEF::HA-Dicer cells. (A) Schematic of endogenously tagged Dicer (HA-Dicer) in primary mouse embryonic fibroblast (PMEF::HA-Dicer) cells (not in scale). HA, epitope tag; DEXD/H, HELICc, helicase domain; DUF283, domain of unknown function; PAZ, Piwi/Argonaute/Zwille; RIIIa/b, RNaseIII a/b; dsRBD, double-stranded RNA binding domain. HA-Dicer is expressed from a single endogenous murine *Dicer* locus by in-frame insertion of a FLAG-HA2-tag upstream of exon 2 as described in [20]. (B) Super-resolution microscopy with 3D projection showing HA (3F10) signals in HA-Dicer cells in absence or presence of Leptomycin B (LMB). Representative images are shown. (C) Quantification of nucleoplasmic (NP) HA signals (3F10) shown as arbitrary values relative to cytoplasm (CP). n>50; asterisk, p<0.05. (D) Immunoblots detecting total HA-Dicer (C29F4, HA.11, 3F10) in subcellular fractions of PMEF::HA-Dicer cells. CP and NP fractions were loaded in a 1:3 ratio. HA signals were quantified using ImageJ. See also section “Materials and Methods” for details on quantitative analysis of nuclear Dicer levels and calculation of % of INPUT values. CTD S2P, RNA polymerase II carboxy-terminal domain (CTD) phospho-residue serine2; Rad21, cohesin subunit; KDEL motif recognising Grp94/Grp78; H3, histone H3.

Comprehensive assessment of subcellular localisation of endogenously tagged HA-Dicer in PMEF::HA-Dicer cells (Fig 1A) critically relies on avoidance of non-physiological artefacts, usage of adequate culture conditions of primary cells and both reliable and sensitive detection methodology. We noticed that low-passaged PMEF::HA-Dicer cells were not dividing as rapidly as wild type PMEF cells. To monitor for potentially elevated levels of senescent cells in our PMEF::HA-Dicer culture, we assessed expression of several proliferation markers following either starvation or serum stimulation (S1 Fig). Expression of cyclin E, cyclin B1, c-Myc and phosphorylation of MAPK effectors ERK1/2 as well as p38 was markedly decreased in PMEF::HA-Dicer cells starved with media containing 0.1% fetal bovine serum (FBS) and restored upon stimulation of starved cells with 20% FBS. In contrast, we could not detected elevated levels of cell cycle inhibitors p21 or p16, irrespective of changes in culture conditions. We conclude that predominantly non-senescent PMEF::HA-Dicer cells were cultured.

**Fig 2:**
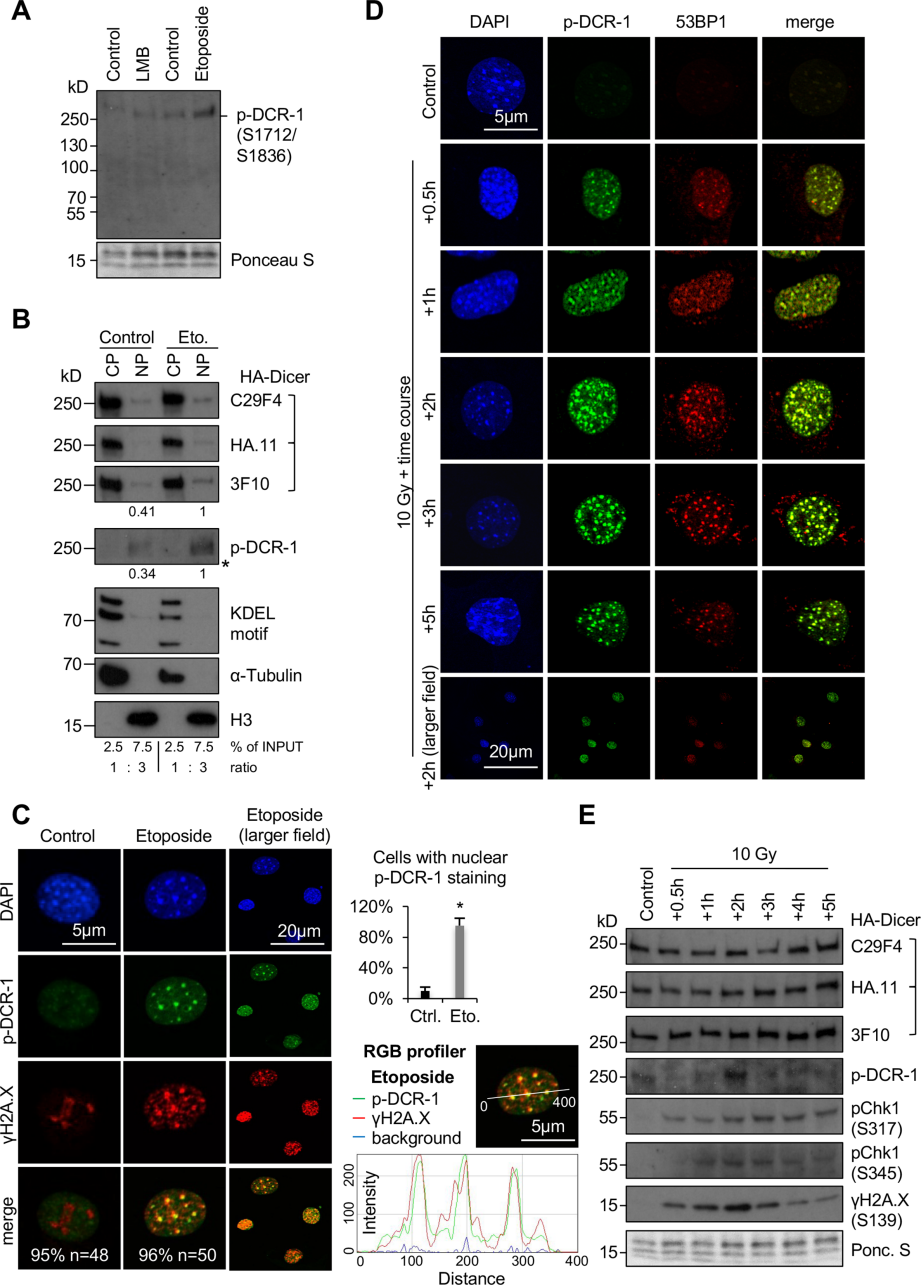
Nuclear localisation of phosphorylated HA-Dicer in damaged PMEF::HA-Dicer cells. (A) Immunoblot displaying reactivity of phospho-Dicer antibodies p-DCR-1 (p-DCR-1, mixture of two individual antibodies recognising Ser1712 or Ser1836 individually, see also section “Materials and Methods” for details) incubated with whole cell extracts of PMEF::HA-Dicer cells following treatment with Leptomycin B (LMB) or Etoposide. Ponceau S, loading control. (B) Immunoblots detecting total (C29F4, HA.11, 3F10) and phosphorylated (p-DCR-1) HA-Dicer in subcellular fractions of PMEF::HA-Dicer cells in absence or presence of Etoposide (Eto.). KDEL motif recognising Grp94/Grp78/protein disulphide isomerase (PDI); H3, histone H3; CP, cytoplasm; NP, nucleoplasm; *, membrane cut. CP and NP fractions are loaded in a 1:3 ratio. See also section “Materials and Methods” for details. (C) Confocal images showing PMEF::HA-Dicer cells stained for phosphorylated HA-Dicer (p-DCR-1) and phosphorylated histone variant H2A.X (γH2A.X, Ser139) in absence or presence of Etoposide. Quantitation indicates cells with shown phenotype in % and number of cell analysed (n). Graph, quantification of cells with p-DCR-1-positive nuclei, n>50; asterisk, p<0.05; RGB profiler, p-DCR-1 (green) and γH2A.X (red) signals in representative cell in presence of Etoposide. (D) Time course confocal imaging of PMEF::HA-Dicer cells stained for phosphorylated Dicer (p-DCR-1) and p53 binding protein 1 (53BP1) following γ-irradiation with a total dose of 10 Gy and recovery. Representative images are shown. (E) Immunoblots detecting total (C29F4, HA.11, 3F10) and phosphorylated (p-DCR-1) HA-Dicer, as well as phosphorylated checkpoint kinase 1 (pChk1, S317/S345) and γH2A.X in PMEF::HA-Dicer whole cell extracts in presence or absence of γ-irradiation (see also S8A Fig for full p-DCR-1 blot). Ponc. S, Ponceau S; loading control.

**Fig 3:**
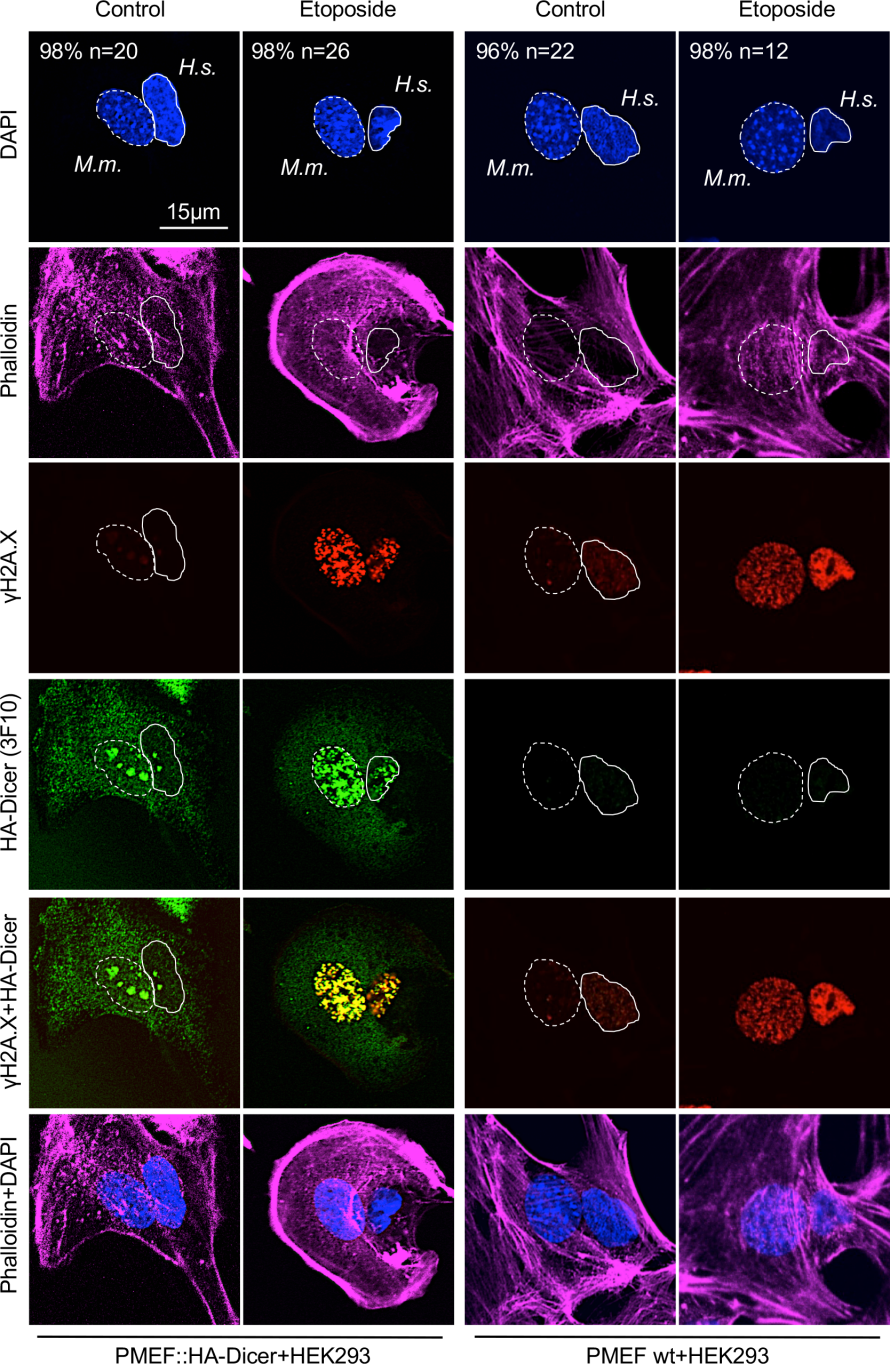
Damage-induced nuclear localisation of murine HA-Dicer in human HEK293 cells. Confocal imaging of interspecies heterokaryon fusions between human HEK293 cells and murine PMEF::HA-Dicer or wild type PMEF cells, respectively. Heterokaryons were stained for total HA-Dicer (3F10), γH2A.X in absence or presence of Etoposide. Cytoskeleton was stained using Alexa Fluor 647-conjugated Phalloidin. Nuclei: *M*.*m*., *Mus musculus*, dashed circle; *H*.*s*., *Homo sapiens*, full circle. n, number of analysed fused cells (n > 15). Representative images are shown.

Previous investigations of HA-Dicer in PMEF::HA-Dicer cells excluded any nuclear localisation or activity of HA-Dicer [19]. We noticed that some imaging data presented in this study displayed a spotted and sporadic distribution of HA antibody signals, not only in the cytoplasm of PMEF::HA-Dicer cells, but also in wild type PMEF nuclei, contrary to a rather homogenous cytoplasmic HA staining of testis, thymus and uterus samples (Figs 1–3 in[19]). Moreover, close inspection of mass spectrometry data provided by Much and colleagues (S1 Table, https://doi.org/10.1371/journal.pgen.1006095.s003) indicates that several factors involved in nuclear RNA metabolism, such as RNA polymerase II co-activators p15 and TIF1B or the pre-mRNA processing factor Fip1 may potentially be overrepresented in HA immunoprecipitations from PMEF::HA-Dicer cells compared to controls from wild type PMEFs, arguably reflecting false-positive enrichment due to non-specific HA antibody reactivity. To optimise conditions for HA-Dicer analysis, control for false-positive data and allow flexibility in antibody combination, we initially tested three different HA epitope tag antibodies, namely rabbit monoclonal C29F4 and mouse monoclonal HA.11, which were both used by Much and colleagues, as well as rat monoclonal 3F10 by immunoblotting. We incubated each antibody with whole cell extracts from either wild type PMEFs, PMEF::HA- Dicer cells or Dicer^-/-^ knockout MEFs (S2A Fig). Each HA antibody generated a prominent band migrating at 250 kD in presence of PMEF::HA-Dicer, but not wild type or Dicer^-/-^ MEF extracts. However, C29F4 generated an additional band, migrating at 130 kD when incubated with either of the extracts. Next, we incubated each HA antibody with serial dilutions of identical PMEF::HA-Dicer extract (S2B Fig). We detected a prominent signal migrating at 250 kD, which was sensitive to dilution, with all three HA antibodies. C29F4 reactivity was lost after diluting the extract 4-fold, whereas HA.11 and 3F10 signals remained detectable at 4-fold dilution. Unlike 3F10, both C29F4 and HA.11 generated additional signals migrating at 130 kD and 80 kD, respectively. To quantify the sensitivity of each HA antibody and visualise HA-Dicer detection thresholds for each HA antibody, we calculated loss of HA reactivity as ratio of relative HA signal normalised to Ponceau S signal from whole cell extract, which we defined as deltaHA (ΔHA), and plotted values over serial dilution steps. We found that at dilution steps 1:2 and 1:4, which allow quantification of signals in the linear range, ΔHA values are highest for C29F4 and lowest for 3F10, indicating increased sensitivity of HA.11 and 3F10 antibodies and compromised specificity of C29F4 and HA.11. We further incubated each HA antibody with whole cell extracts from PMEF::HA-Dicer following starvation or serum stimulation (S2C Fig). Again, C29F4 and HA.11, but not 3F10, generated aberrant bands of high molecular weight, which were sensitive to starvation and induced by serum stimulation. Of note, it is currently unclear, whether aberrant bands detected with HA antibodies C29F4 and HA.11 reflect predominantly unspecific signals or may also display Dicer cleavage products with potential relevance for Dicer localisation and function.

Next, we tested for specificity and sensitivity of HA antibodies in immunofluorescence microscopy. We co-cultured wild type PMEFs and PMEF::HA-Dicer cells in absence or presence of nuclear export inhibitor LMB or DNA damage-inducing Topoisomerase II inhibitor Etoposide prior to HA staining with either C29F4, HA.11 or 3F10 antibody (S3A Fig.). Each HA antibody generated prominent cytoplasmic reactivity in a subset of untreated control cells, arguably reflecting HA-Dicer expressed in PMEF::HA-Dicer cells. Cytoplasmic HA reactivity was accompanied by increased nuclear HA staining upon treatment with LMB or Etoposide. Importantly, treatment with LMB or Etoposide neither caused significant onset of nuclear HA reactivity in cells without cytoplasmic HA staining, indicating specificity of each individual HA antibody in confocal imaging (S3A Fig). Drug treatments also did not alter expression of full length HA-Dicer (S3B Fig). Again, we detected several aberrant signals when probing with C29F4 and HA.11, but not 3F10 HA antibody. We conclude that the 3F10 HA antibody is most specific and sensitive for detection of HA-Dicer by immunoblotting, whereas differences among HA antibodies seem marginal when used in confocal imaging.

To reassess the subcellular localisation of HA-Dicer in unperturbed PMEF::HA-Dicer cells, we combined the 3F10 HA antibody with highly sensitive super-resolution microscopy. We detected clear nuclear 3F10 staining, in addition to prominent reactivity in the cytoplasm (Fig 1B). Incubation with LMB enhanced nuclear HA-Dicer 3F10 signals 2-3-fold (Fig 1C). To substantiate nuclear HA-Dicer localisation, we applied subcellular fractionation and probed for HA-Dicer with each individual HA antibody (Fig 1D). Although the bulk of HA-Dicer was present in the cytoplasmic fraction, we detected a clear HA-Dicer signal in the nuclear fraction using C29F4 or HA.11 antibodies. The 3F10 antibody generated the strongest nuclear HA-Dicer signal. Importantly, cytoplasmic and endoplasmatic reticulum (ER) membrane-associated contaminants were not detectable in nuclear fractions.

To estimate the amount of nuclear HA-Dicer in the nucleus of unperturbed cells quantitatively, we measured nuclear HA-Dicer band intensities relative to cytoplasmic levels and calculated the amount of nuclear Dicer in % normalised to a cytoplasmic-to-nucleoplasmic input ratio of 1:3, reflecting a 3-fold concentrated nuclear fraction. Values for nuclear HA-Dicer varied between 3.7% and 9.7%, depending on the HA antibody. We conclude that a subset of approximately 5% of the total HA-Dicer pool localises to the nucleus of PMEF::HA-Dicer cells under physiological conditions.

We have recently discovered that a subset of human Dicer is phosphorylated in response to DNA damage to process damage-induced dsRNA in the nucleus [24] and wished to assess murine HA-Dicer subcellular localisation in context of DNA damage. First, we monitored that PMEF::HA-Dicer cells are responsive to DNA damage. We detected elevated levels Ataxia telangiectasia mutated (ATM)/ATM-related (ATR) kinase substrates, increased phosphorylation of histone variant H2A.X (γH2A.X) and induction of downstream effector p21, indicating onset of DNA damage signalling upon treatment with γ-irradiation and Etoposide, but not with LMB (S4A Fig). Next, we made use of two recently described phospho-specific Dicer antibodies, which were raised against two conserved carboxy-terminal murine Dicer phospho-serine residues Ser1712 and Ser1836 [26]. We used a mixture of both phospho-antibodies, hereinafter p-DCR-1 antibodies, for comprehensive detection of phosphorylated HA-Dicer. Of note, we have previously confirmed the specificity of p-DCR-1 antibodies by mutation of both phospho-serine epitopes into alanine residues in human HEK293 cells [24]. When incubating p-DCR-1 antibodies with PMEF::HA-Dicer whole cell extracts, we detected increased p-DCR-1 reactivity following preincubation with Etoposide, but not LMB (Fig 2A). To further validate specificity of p-DCR-1 antibodies, we used the 3F10 HA antibody to immunoprecipitate comparable amounts of total HA-Dicer from PMEF::HA-Dicer, but not wild type PMEF cells cultured in presence or absence of Etoposide (S4B Fig.). Elevated levels of γH2A.X were indicative for Etoposide-induced DNA damage. When probing immuno-selected samples with p-DCR-1 antibodies, we detected faint, but modestly elevated reactivity upon Etoposide treatment (S4C Fig). Moreover, p-DCR-1 reactivity was detectable when probing whole cell extracts from wild type PMEF and PMEF::HA-Dicer cells, but not Dicer^-/-^ MEFs upon incubation with Etoposide (S4D Fig.). We conclude that p-DCR-1 antibodies specifically detect damage-induced Dicer phosphorylation on immunoblots.

Next, we preformed subcellular fractionation of PMEF::HA-Dicer cells cultured in presence or absence of Etoposide (Fig 2B). Using HA antibodies C29F4 or HA.11, we found the bulk of the total HA-Dicer pool localising in the cytoplasm, with a small fraction present in nuclei, irrespective of DNA damage. In contrast, signals for both total and phosphorylated HA-Dicer were elevated 2-3-fold in damaged nuclei upon detection with 3F10 and p-DCR-1 antibodies. We could, however, not detect a shift in migration of HA-Dicer, indicating that the DDR targets a relatively small number of Dicer molecules. We noticed that Much and colleagues failed to detect nuclear HA-Dicer in subcellular fractions and speculated that this may be due to different amounts of material loaded as nuclear input, which does not include 3-fold concentrated nuclear samples. Indeed, when loading fractions in a 1:1 ratio, we could not detect clear HA-Dicer signals in nuclear fractions on blots displaying prominent cytoplasmic HA-Dicer levels, irrespective of Etoposide-induced DNA damage (S4E Fig).

To visualise phosphorylated HA-Dicer, we performed confocal imaging of PMEF::HA-Dicer cells stained with p-DCR-1 antibodies in absence or presence of Etoposide (Fig 2C). Strikingly, we detected formation of nuclear, p-DCR-1-positive foci in >90% of cells incubated with Etoposide, but not in undamaged control nuclei and confirmed partial colocalisation of p-DCR-1 signals with γH2A.X-positive damage foci using RGB profiler. To confirm that Etoposide-induced nuclear HA-Dicer localisation is primarily caused by induction of DSBs, we tested for formation of p53 binding protein 1 (53BP1)-positive damage foci, a hallmark of DSB repair [27]. Indeed, we detected strong nuclear 53BP1 staining in damaged cells and partial colocalisation of 53BP1 signal with nuclear HA-Dicer upon Etoposide incubation (S4F and S4G Fig). Next, we wished to control for specific detection of total and phosphorylated HA-Dicer in immunofluorescence microscopy. Therefore, we co-cultured a mixture of both wild type PMEFs, PMEF::HA-Dicer cells and Dicer^-/-^ MEFs in absence or presence of Etoposide and co-stained cells with HA (3F10) and p- DCR-1 antibodies (S4H Fig). In absence of Etoposide, strong reactivity of HA, but not p- DCR-1 antibodies was detected predominantly in the cytoplasm in a subset of cells. Treatment with Etoposide, however, induced additional nuclear HA staining and onset of nuclear p-DCR-1 reactivity in cells both positive and negative for HA staining. Importantly, a subset of cells remained negative for both HA and p-DCR-1 reactivity, indicating that p-DCR-1 antibodies detected phosphorylated HA-Dicer in damaged wild type PMEF and PMEF::HA-Dicer, but not Dicer^-/-^ MEF cells.

Three members of the phosphatidylinositol-3-kinase (PI3K) family—ATM, ATR and DNA-dependent protein kinase (DNA-PK)—govern the response to DNA damage by phosphorylating hundreds of substrates [28–31]. To investigate the contribution of PI3Ks to damage-induced phosphorylation of murine HA-Dicer, we treated PMEF::HA-Dicer cells with Etoposide in absence or presence of PI3K inhibitors and imaged cells using p-DCR-1 and HA antibodies (S5A Fig). Again, we detected prominent nuclear localisation of p-DCR-1 foci and HA-Dicer in the vast majority of damaged nuclei. In contrast, preincubation of PMEF::HA-Dicer cells with PI3K inhibitors prior to Etoposide treatment attenuated both p-DCR-1 and HA staining in the nucleus in 80–90% of imaged cells. When monitoring for HA-Dicer expression levels in absence or presence of Etoposide or upon preincubation with PI3K inhibitors we could not detect significant alterations in HA-Dicer levels (S5B Fig). To control for activity of PI3K inhibitors, we probed for phosphorylation of downstream targets checkpoint kinase 1 (Chk1) and γH2A.X. We confirmed that inhibition of ATR or ATM impairs Etoposide-induced phosphorylation of Chk1 or H2A.X, respectively. We conclude that phosphorylated HA-Dicer accumulates in the nucleus in response to DNA damage in a PI3K-dependent manner.

To further assess the subcellular localisation of phosphorylated HA-Dicer in PMEF::HA-Dicer cells in response to DNA damage, we preformed γ-irradiation. We have recently described damage-induced nuclear localisation of phosphorylated human Dicer 2–3 hours after γ-irradiation with a total dose of 10 Gray (Gy) [24]. To assess HA-Dicer localisation in response to γ-irradiation under optimised conditions, we employed a time course experiment using a total dose of 10 Gy γ-irradiation followed by up to 5 hours recovery (Fig 2D). Formation of 53BP1-positive foci was used as a marker for DSBs. We detected a wave of p-DCR-1 reactivity, which was detectable concomitantly with induction and clearance of 53BP1-positive damage foci. We found that 53BP1 foci formation was induced 30 minutes after irradiation modestly, but peaked 2 hours after irradiation and was reduced after 5 hours. The p-DCR-1 signal was also modestly detectable after 30 minutes, but most clearly detectable 2 hours after irradiation. We further confirmed formation of DSBs by detection of prominent γH2A.X -positive foci, which partially co-localised with p-DCR-1 staining (S6 Fig). Moreover, prominent nuclear localisation of total HA-Dicer was also detectable 2 hours after irradiation using 3F10 HA antibody, suggesting recruitment of phosphorylated HA-Dicer to close proximity of DSBs.

To further validate specificity of 3F10 HA and p-DCR-1 antibodies in confocal imaging, we repeated γ-irradiation in both wild type PMEFs and Dicer^-/-^ MEFs at optimised conditions. As expected, γ-irradiation neither induced 3F10 reactivity in wild type PMEFs (S7A Fig), nor p-DCR-1 reactivity in Dicer^-/-^ MEFs (S7B Fig), nor reactivity of secondary Alexa Fluor antibodies (S7C Fig). In analogy to Etoposide treatment, we repeated co-culture of wild type PMEF wild type PMEFs, PMEF::HA-Dicer cells and Dicer^-/-^ MEFs and stained for HA-Dicer using HA and p-DCR-1 in presence of γ-irradiation (S7D Fig.). Again, we detected increased nuclear HA reactivity in a subset of irradiated cells and onset of nuclear p-DCR-1 reactivity in cells both positive and negative for HA staining as well as cells, which were negative for pDCR-1 and HA signals, despite being irradiated. Much and colleagues used γ-irradiation to study HA-Dicer subcellular localisation in response to DNA damage at a single time point, namely 30 minutes after irradiation with a total dose of 20 Gy, but failed to detect nuclear HA-Dicer localisation. We speculated that suboptimal conditions were used to induce phosphorylation of HA-Dicer by DNA damage signalling in PMEF::HA-Dicer cells. Surprisingly, we detected prominent reactivity for both total and phosphorylated HA-Dicer in damaged nuclei as little as 30 minutes after high dose irradiation (S7D Fig.). Importantly, we further confirmed induction of HA-Dicer phosphorylation by γ-irradiation and specificity of p-DCR-1 antibodies by (i) immunoblotting with p-DCR-1 antibodies with irradiated wild type PMEF, PMEF::HA-Dicer and Dicer^-/-^ MEF whole cell extracts (S8A Fig.), (ii) partial colocalisation of p-DCR-1 and HA signals upon γ-irradiation using RGB profiling (S8B Fig.) and (iii) by super-resolution imaging followed by 3D reconstitution (S8C Fig.). Modest colocalisation of p-DCR-1 and HA signals could also be observed in cells analysed immediately after irradiation (30 minutes), but not after prolonged incubation (5 hours) or non-irradiated control cells, suggesting that a threshold of DNA damage limits detection of nuclear phosphorylated HA-Dicer following irradiation with 10 Gy. Reassuringly, γ-irradiation induced phosphorylation of DNA damage signalling components Chk1 and H2A.X, but did not significantly alter HA-Dicer levels over time (Fig 2E). Of note, detection of nuclear phosphorylated HA-Dicer appeared to be clearer in immunofluorescence microscopy than on immunoblots. The reason for this apparent discrepancy is currently unclear, but might at least in part be due to intrinsic differences in p-DCR-1 antibody sensitivities, as p-DCR-1 antibodies are primarily suited for immunofluorescence microscopy and comprise limited performance in immunoblotting (Swathi Arur, personal communication).

For proof of principle, we performed an interspecies heterokaryon experiment (Fig 3). Sporadic formation of interspecies heterokaryons containing both murine and human nuclei was confirmed by DAPI staining, displaying typical spotted murine nuclei, and staining with Phalloidin, displaying a cellular continuum. Strikingly, we detected strong, nuclear 3F10 HA antibody reactivity in HEK293 cells fused to PMEF::HA-Dicer, but not wild type PMEF cells in Etoposide-treated cells. Induction of nuclear 3F10 reactivity occurred concomitant with formation of γH2A.X-positive DNA damage foci and partial colocalisation with γH2A.X. As only PMEF::HA-Dicer cells express HA-tagged Dicer, we conclude that the HA signal detectable in HEK293 nuclei originated in PMEF::HA-Dicer cells. Minor nuclear HA reactivity was also detectable in HEK293 cells fused to PMEF::HA-Dicer in absence of Etoposide, arguably reflecting non-specific accumulation of HA-Dicer in nucleoli after permeabilisation and displacement of ribosomal RNA. Importantly, however, no HA signal was detectable in HEK293 cells fused to wild type PMEFs, irrespective of Etoposide treatment, underscoring specificity of the 3F10 HA antibody toward HA-Dicer and damage-induced nuclear accumulation of HA-Dicer.

Taken together, our data indicate that a subset of HA-Dicer localises to nuclei of unperturbed cells, with increased levels of phosphorylated HA-Dicer being detectable in damaged nuclei. We show that phosphorylation of HA-Dicer in response to DNA damage triggers accumulation of HA-Dicer in the nucleus. Phosphorylated nuclear HA-Dicer arguably reflects a minor fraction of the HA-Dicer pool, which we estimate to be 5%. Importantly, no adverse impact on viability and fertility of mice homozygous for the *Dcr*^*FH*^ allele has been reported, suggesting that HA-Dicer is not compromised in its localisation or function by introduction of the HA epitope tag *per se* [20]. Advancing on previous Dicer localisation studies, our findings provide insight into nuclear Dicer localisation and function under physiological conditions. Since we monitor for specificity and sensitivity of HA-Dicer detection using various controls, we conclude that Much and colleagues may have failed to detect changes in the subcellular localisation of murine HA-Dicer upon DNA damage or other stimuli due to technical limitations, such as lack of antibody sensitivity.

Recent evidence from *C*. *elegans* Dicer, DCR-1, demonstrates that phosphorylated DCR-1 is accumulating and functional in nuclei. In the adult worm germ line, DCR-1 localises in uniformly distributed cytoplasmic and nuclear foci and on the inner side of nuclear pores [32]. During development, the Ras-dependent MAP kinase MPK-1, a homologue of ERK kinases in mammals, phosphorylates cytoplasmic DCR-1 at two serine residues in the C-terminal RNase III and dsRBD domains, which triggers nuclear translocation of phosphorylated DCR-1 [26]. A similar translocation phenotype has been observed in human HEK293 cells. Importantly, phosphorylation of conserved C-terminal residues by MAPK signalling is conserved in mammalian Dicer, as demonstrated by *in vitro* kinase assays and fibroblast growth factor (FGF) stimulation [26]. Along the same lines, we showed accumulation of human phosphorylated Dicer in damaged nuclei, and discovered the damage-inducible Dicer residue serine-1016, which facilitates accumulation of Dicer in the nucleus and processing of nuclear, damage-induced dsRNA [24].

We conclude that Dicer proteins are found in the nuclei of the vast majority of studied eukaryotes, including mammals. The cytoplasm remains the main compartment for Dicer localisation. However, during development or stress, a subset of the cytoplasmic Dicer pool may be altered either genetically, by proteolysis, heat shock, or by PTMs to adjust for Dicer subcellular localisation or activity, suggesting structural and functional distinct nuclear Dicer subpopulations. Our findings point toward additional layers of complexity in the regulation of RNAi components and underscore the relevance of studying mechanisms of non-canonical RNAi in mammals.

## Materials and methods

### Tissue culture, cell lines and inhibitors

Wild type or HA epitope-tagged primary mouse embryonic fibroblasts (PMEF wt and PMEF::HA-Dicer, female, a kind gift from the O’Carroll Lab), or Dicer knockout mouse embryonic fibroblasts (MEF Dicer^-/-^, clone [1A11], [4] a kind gift from the Heissmeyer Lab), or wild type human HEK293 cells were cultured in Dulbecco's modified Eagle complete medium (DMEM, Sigma) supplemented with 10% fetal bovine serum (FBS, Life Technologies), 2 mM L-glutamine, 1x non-essential amino acids and 100 units/ml penicillin/streptomycin at 37°C and 5% CO_2_ at low passages (<20 passages). Non-immortalized PMEFs were derived from E13.5 embryos of *Dcr*^*FH/+*^ intercrosses according to standard protocols. For serum starvation, PMEF::HA-Dicer cells were shifted to DMEM containing 0.1% FBS for 24 hours. For serum stimulation, PMEF::HA-Dicer cells were starved in DMEM containing 0.1% FBS for 20 hours, followed by incubation with DMEM containing 20% FBS for 4 hours prior to lysis. Nuclear export was inhibited with CRM1/exportin1-inhibitor Leptomycin B (Cayman, 20 nM, 16 hours). DNA damage was induced with the Topoisomerase II inhibitor Etoposide (Sigma, 25 μM, 2 hours) or γ-irradiation using at total dose of 10 or 20 Gy. Small-molecule inhibitors KU-55933 (ATM inhibitor, 5 μM, Sigma); VE-821 (ATR inhibitor, 1 μM, Sigma); LY294002 (PI3K inhibitor, 5 μM, NEB) were used for 1 hour prior to induction of DNA damage.

### Subcellular fractionation, immunoprecipitation and immunoblotting

Subcellular fractionation was performed as described [10]. Approximately 3x 10^6^ PMEF::HA-Dicer cells grown on 10 cm dishes (Corning), were trypsinised, washed in cold 1x PBS and centrifuged (1200rpm, 5 min). Pellets were lysed in five volumes (i.e. 300 μl) of hypotonic lysis buffer (10 mM HEPES pH 7.9, 60 mM KCl, 1.5 mM MgCl_2_, 1 mM EDTA, 1 mM DTT, 0.075% NP-40, 1x protease/phosphatase inhibitor cocktails, Roche) and incubated for 10 minutes at 4°C with rotation. Nuclei were pelleted by centrifugation (1200 rpm, 4°C) for 10 minutes. The cytoplasm was collected from the supernatant. Nuclei were washed five times in 800 μl hypotonic lysis buffer without NP-40 and lysed in 1 volume (i.e. 33 μl) of nuclear lysis buffer (20 mM HEPES pH 7.9, 400 mM NaCl, 1.5 mM MgCl_2_, 0.2 mM EDTA, 1 mM DTT, 5% Glycerol, 1x protease/phosphatase inhibitor cocktails, Roche). Lysates were diluted with two volumes (i.e. 66 μl) of dilution buffer (20 mM HEPES pH 7.9, 1.6% Triton- X-100, 0.2% Sodium deoxycholate, 1x protease/phosphatase inhibitor cocktails, Roche), followed by 10 sec sonication with a Bioruptor (Diagenode) at low energy and incubation with 10 U Benzonase (Sigma) for 5 min. Lysates were centrifuged (13500 rpm, 4°C, 10 minutes) and the supernatant (i.e. 100 μl) was collected as soluble nuclear fraction. 10% of subcellular fractions (i.e. 30 μl) of cytoplasmic and 10 μl of nuclear fraction were boiled in 0.25x volume (i.e. 30 μl for cytoplasm or 3.33 μl for nuclei) of 4 x SDS-PAGE sample buffer (12% SDS, 40 mM Tris HCl pH 7.4, 40% glycerol, 3% beta-Mercaptoethanol, 1% Bromophenol Blue) at 95°C for 5 minutes, respectively. Samples were sonicated and 10 μl of either cytoplasmic or nuclear fractions (i.e. 25% or 75% of boiled samples, respectively), were analysed by Western Blot using precast gels (Mini-PROTEAN TGX, BioRad). Each gel lane was loaded with 1/40 (10% x 25%, i.e. 2.5% of lysed cytoplasm) or 3/40 (10% x 75%, i.e. 7.5% of lysed nuclei), respectively, in a 1:3 ratio, unless stated differently.

The amount of nuclear Dicer relative to cytoplasmic levels and normalised to the 1:3 input ratio was calculated in % using the following equation: [(HA Ab signal in nuclear fraction / HA Ab signal in cytoplasmic fraction) / (7.5% of lysed nuclei / 2.5% of lysed cytoplasm)] x 100%. Intensities of bands were quantified using ImageJ and values for nuclear Dicer were plotted as relative signals normalised to signals from cytoplasmic fractions or non-damaged nuclear fractions, respectively. For example, calculation of amount of nuclear Dicer using 3F10 Ab as shown in Fig 1D: [(0.29/1) / (7.5 / 2.5)] x 100% = 9.7%. Values for nuclear Dicer using C29F4 or HA.11 were 8.0% or 3.7%, respectively.

Whole cell extracts from approximately 5x 10^5^ cells grown on 6-well multi-well dishes (Corning) were lysed directly in 100 μl 4 x SDS-PAGE sample buffer, 10 μl of lysate was loaded, separated and stained with Ponceau S (Sigma) prior to antibody hybridisation. For semi-quantitative analysis of detection thresholds of HA antibodies, 10 μl lysate of PMEF::HA-Dicer cells, which were grown on 6-well multi-well dishes and lysed in 100 μl 4 x SDS-PAGE sample buffer, was diluted 5 times in a 2-fold serial dilution series. HA signal intensities of 10 μl of either non-diluted sample, i.e. input, or diluted samples, or Ponceau S stainings thereof, were quantified using ImageJ and plotted as relative normalised signals. Signals were plotted, loss of reactivity of HA antibodies was visualised as gap and quantified as delta (Δ = Rel. norm. Ponc. S signal—Rel. norm. HA Ab signal). For each HA antibody, a Δ was calculated at dilutions steps 1:2 or 1:4 to quantify signals in the near-linear range of sensitivity.

For immunoprecipitation, approximately 3x 10^6^ wild type PMEF or PMEF::HA-Dicer cells grown on 10 cm dishes (Corning) were trypsinised, washed in cold 1x PBS and centrifuged (1200rpm, 5 min). Pellets were lysed in 5 volumes WCE lysis buffer (20 mM Tris pH 7.5, 150 mM NaCl, 0.1% NP-40, 2 mM MgCl_2_, 50 mM NaF, 1 x protease/phosphatase inhibitor cocktails, Roche) for 20 minutes on ice, sonicated and Benzonase digested as described above. WCE lysates were precleared with protein G agarose beads (Merck Millipore) for 30 min. Samples were incubated with 5 ug primary HA antibodies for 4 hours and pulled down using protein G agarose beads for 45 min. IP samples were washed three times for 10 min with 800 μl WCE lysis buffer (20 mM Tris pH 7.5, 150 mM NaCl, 0.1% NP-40, 2 mM MgCl_2_, 50 mM NaF, 1 x protease/phosphatase inhibitor cocktails, Roche), and eluted with SDS-PAGE sample buffer.

The following primary antibodies were used: anti-α-Tubulin (Abcam, [YL1/2], ab6160); anti-Rad21 (Merck Millipore, 05–908); anti-RNAPII-CTD S2P (Abcam, ab5095); anti-histone H3 (Abcam, ab1791); anti-HA (Roche, [3F10], 11867423001); anti-HA (BioLegend, [16B12], 901501, previously Covance MMS-101P); anti-HA (CST, [C29F4], 3724); anti-Lamin C (Novus, [EM11], NBP1-50051); anti-KDEL motif (Abcam, [10C3], ab12223); anti-γH2A.X (Ser139, Merck Millipore, 05–636); anti-Drosha (Abcam, ab12286); anti-Cyclin E (Santa Cruz, [M-20], sc-481); anti-Cyclin B1 (Abcam, ab2949); anti-c-Myc (Clontech, 631206); anti-53BP1 (Santa Cruz, [H-300], sc-22760); anti-pATM/ATR substrates mix (CST, [SxQ, D23H2/D69H5], 9670); anti-pChk1 (Ser345, CST, 133D3); anti-pChk1 (Ser317, CST, D12H3); anti-pERK1/2 (Thr202/Tyr204, CST, 9101); anti-ERK1/2 (CST, 9102); anti-p-p38 (Thr180/Tyr182, CST, 9211); anti-p21 (Santa Cruz, [C-19], sc-397); anti-p16 (Santa Cruz, [F-12], sc-1661); and anti-p-DCR-1 (Ser1712/Ser1836, a kind gift from the Arur Lab) [26].

The p-DCR-1 signals represent a mixture of two individual antibodies, raised against carboxy-terminal murine Dicer epitopes phospho-Ser1712 and phospho-Ser1836 individually in separate rabbits. Murine epitopes Ser1712/Ser1836 are equivalent to human epitopes Ser1728/Ser1852 and *C*. *elegans* epitopes Ser1705/Ser1833. However, human and mouse epitopes differ by one amino acid relative to the original epitope in *C*. *elegans*.

### Imaging analysis

Approximately 3x 10^5^ wild type PMEFs, Dicer^-/-^ MEFs clone [1A11], and PMEF::HA-Dicer cells grown on 6-well multi-well dishes (Corning) were washed in 1x PBS, fixed on coverslips with 3% Paraformaldehyde in PBS for 10 min, washed and incubated with 50 mM Ammonium chloride in PBS for 10 min, washed in 1x PBS, permeabilised with PBS/0.1% Tween for 5 min and blocked with PBS/10% FBS for 2 hours at 4°C. Primary antibodies were incubated overnight at 4°C in PBS/0.15% FBS. Alexa Flour 488-, 555-, or 647-conjugated secondary antibodies (Invitrogen) were incubated in PBS/0.15% FBS at room temperature for 1.5 hours in a humidified chamber. Cells were washed 3 times for 5 minutes with PBS/0.1% Triton-X 100 between antibody incubations. Nuclei were counterstained and mounted with 6-diamidino-2-phenylindole (DAPI)-containing Mowiol (Merck Millipore).

For confocal imaging, slides were processed on an Olympus microscope, using 60x lens. Samples with 1.5-mm coverslips were imaged using a an FV1000 confocal system on an Olympus IX-81 microscope with photomultiplier tube detectors and Olympus PlanApo N, 60×/1.35NA lens at RT. DAPI-containing Mowiol (EMD Millipore) was used as the imaging medium. DAPI; Alexa Fluor 488, 539, and 635 (Thermo Fisher Scientific) channels were used for acquisition with Olympus Fluoview software. Experimental settings including values of the laser power for each channel, HV, gain and offset parameters were determined at the beginning of each individual imaging session (by assessing background reactivity and saturation levels of each channel) and kept constant over the entire imaging session. ImageJ software (NIH) was used for further processing of the images. All quantifications represent a number of cells that have shown phenotype or % of positive cells, see figure legends for details. n = number of cells. Signals were quantified using RGB profiler (ImageJ, NIH). For super-resolution microscopy, cells were imaged at room temperature using an inverted Zeiss 880 microscope fitted with an airy-scan detector. The system was equipped with Plan- Apochromat 63x/1.4 NA oil lens. 488 nm argon and 405nm, and 633 nm diode lasers were used to excite GFP, DAPI and Alexa Fluor 633, respectively. Sequential excitation of each wavelength was switched per line to ensure blue, green and red channels were aligned. Sections of 20 slices with 0.5 μm thick intervals were collected with a zoom value of 600 pixels/μm. Images were processed using Airyscan processing (Zeiss 880 Airyscan: Airyscan is a special detector added to the coupling port of LSM 880. It is more light efficient than a standard confocal point detector. The extra on photons can be used to increase the sensitivity of the image, to scan faster or to improve the resolution) in 3D with a strength value of *Auto* (~6). 3D representation were generated using Imaris 8.4.1 (Bitplane, Oxford Instruments).

### Heterokaryon formation

Approximately 3x 10^5^ murine wild type PMEF or PMEF::HA-Dicer cells were grown to 70–80% confluency on 6-well multi-well dishes (Corning). Approximately 2x 10^5^ wild type human HEK293 cells were seeded on top of the PMEF layer prior to membrane fusion. Mixed cell populations were grown in presence of Cycloheximide (50 μg/ml) for 4 hours prior to fusion. For heterokaryon formation, cells were washed with warm 1x PBS, incubated with 100 μl warm PEG-3000 solution (50% w/v in PBS) for 2 minutes and washed with 1x PBS five times. Heterokaryons were cultured for additional 2 hours in Cycloheximide- containing medium in presence or absence of Etoposide (25 μM) prior to fixation. Alexa Fluor 647-conjugated Phalloidin (Life Technology) was used to stain the cytoskeleton. Preparation of immunofluorescence slides was performed as described in section imaging analysis.

## Supporting information

S1 Fig PMEF::HA-Dicer cells are responsive to serum stimulation. Immunoblots probing for cyclin E, cyclin B1, c-Myc, as well as total and phosphorylated ERK1/2 kinases, phosphorylated p38 kinase, or cell cycle inhibitors p21 and p16 in PMEF::HA-Dicer whole cells extracts following starvation (0.1% fetal bovine serum, FBS) or serum stimulation (0.1% FBS +20% FBS). Control, 10% FBS; Rad21, cohesin subunit, loading control; #, unspecific signal.
(TIF)

S2 Fig Validation of HA antibodies C29F4, HA.11 and 3F10. (A and B) Immunoblots displaying reactivity of HA antibodies C29F4, HA.11, 3F10 following incubation with (A) whole cell extracts of wild type PMEF, PMEF::HA-Dicer or Dicer^-/-^ knockout MEF cells (clone 1A11) and with (B) serial dilution of PMEF::HA-Dicer whole cell extract. HA signals were quantified as arbitrary units relative to Ponceau S staining and normalised to input (IN, undiluted whole cell extract, 10% of lysate is loaded). Loss of reactivity of HA antibodies is defined as deltaHA (ΔHA = Rel. norm. Ponc. S signal—Rel. norm. HA Ab signal). Values at non-diluted input samples were set to 1. Ponceau S, loading control; #, aberrant signals; Ab, antibody. See also section “Materials and Methods” for details. (C) Immunoblots detecting total HA-Dicer (C29F4, HA.11, 3F10) in PMEF::HA-Dicer whole cells extracts following starvation (0.1% FBS) or serum stimulation (0.1% FBS +20% FBS). Control, 10% FBS; Rad21, cohesin subunit, loading control; #, aberrant signals.
(TIF)

S3 Fig Nuclear accumulation of HA-Dicer in PMEF::HA-Dicer cells upon nuclear export inhibition or DNA damage. (A) Confocal imaging of wild type PMEF and PMEF::HA-Dicer co-cultures using HA antibodies C29F4, HA.11 and 3F10 in absence or presence of Leptomycin B (LMB) or Etoposide. Representative merged images are shown.
(B) Immunoblots displaying reactivity of HA antibodies C29F4, HA.11 and 3F10 incubated with whole cell extracts of PMEF::HA-Dicer cells following treatment with Leptomycin B (LMB) or Etoposide. #, aberrant signals; Ab, antibody.
(TIF)

S4 Fig Phosphorylation of HA-Dicer in PMEF::HA-Dicer cells. (A) Immunoblots detecting substrates of Ataxia-telangiectasia mutated (ATM) and ATM-related (ATR) kinase activity (pATM/ATR substrates mix antibody), p21 and phosphorylated histone variant H2A.X (γH2A.X, Ser139) levels in PMEF::HA-Dicer whole cell extracts following γ-irradiation (10 Gy, 2 hours recovery time) or incubation with Etoposide or Leptomycin B (LMB). (B) Immunoblots displaying reactivity of HA antibody 3F10 and γH2A.X levels after incubation with whole cell extracts (IN, input, 10% of lysate is loaded) of wild type PMEFs or PMEF::HA-Dicer cells or after immunoprecipitation (IP) using 3F10 antibody in absence or presence of Etoposide. IgG, immunoglobulin heavy chain, loading control. (C) Immunoblot detecting phosphorylated HA-Dicer using p-DCR-1 antibodies following IP with 3F10 antibody as described in (B); #, aberrant signal; M, molecular-weight size marker. (D) Immunoblots displaying reactivity of p-DCR-1 antibodies following incubation with whole cell extracts of wild type PMEFs, PMEF::HA-Dicer cells or Dicer^-/-^ knockout MEFs after treatment with Etoposide. H3, histone 3, loading control; #, unspecific signal. (E) Immunoblots detecting total HA-Dicer (HA.11, 3F10) in subcellular fractions of PMEF::HA- Dicer cells in absence or presence of Etoposide. H3, histone H3; CP, cytoplasm; NP, nucleoplasm; CP and NP fractions are loaded in a 1:1 ratio. See also section “Materials and Methods” for details. (F and G) Confocal images showing PMEF::HA-Dicer cells stained for p53 binding protein 1 (53BP1) and total HA-Dicer using HA antibody 3F10 (F) or HA.11 (G) in absence or presence of Etoposide. Representative images are shown. (H) Confocal imaging of wild type PMEF (*wt*), PMEF::HA-Dicer (*HA*) and Dicer^-/-^ MEF (*-/-*) co-cultures using 3F10 and p-DCR-1 antibodies in absence or presence of Etoposide. Representative merged images are shown.
(TIF)

S5 Fig Phosphatidylinositol-3-kinase (PI3K)-dependent nuclear localisation of HA- Dicer in PMEF::HA-Dicer cells. (A) Confocal imaging of phosphorylated HA-Dicer (p-DCR-1) and total HA-Dicer (3F10) in PMEF::HA-Dicer cells in presence or absence of Etoposide or after preincubation with Phosphatidylinositol-3-kinase (PI3K) inhibitors. Quantitation indicates cells with shown phenotype in % and number of cell analysed (n) (top panel). RGB profiles of p-DCR-1 (green) and HA-Dicer (red and blue) signals in representative cells. (B) Immunoblots detecting total HA-Dicer (3F10, HA.11, C29F4) as well as levels of phosphorylated checkpoint kinase 1 (pChk1, Ser317/Ser345) and γH2A.X in PMEF::HA-Dicer cells in presence or absence of Etoposide or after preincubation with Phosphatidylinositol-3-kinase (PI3K) inhibitors. Ponceau S, loading control.
(TIF)

S6 Fig Accumulation of nuclear phosphorylated HA-Dicer at γH2A.X-positive damage foci in PMEF::HA-Dicer cells upon γ-irradiation. Time course confocal imaging of PMEF::HA-Dicer cells stained for phosphorylated Dicer (p-DCR-1), γH2A.X and total HA-Dicer (3F10) following γ-irradiation with a total dose of 10 Gy and recovery for various hours. Quantitation indicates cells with shown phenotype in % and number of cell analysed (n).
(TIF)

S7 Fig Validation of 3F10 HA epitope and p-DCR-1 antibodies. (A) Confocal imaging of wild type PMEF cells stained for phosphorylated Dicer (p-DCR-1), γH2A.X and total HA- Dicer (3F10) following γ-irradiation with a total dose of 10 Gy and 2 hours recovery time.
(B) as in (A), but using Dicer^-/-^ MEFs (clone 1A11). (C) Confocal imaging of PMEF::HA- Dicer cells incubated with Alexa-Flour conjugated secondary antibodies. (D) Confocal imaging of wild type PMEF (*wt*), PMEF::HA-Dicer (*HA*) and Dicer^-/-^ MEF (*-/-*) co-cultures using 3F10 and p-DCR-1 antibodies following γ-irradiation with a total dose of 20 Gy and 2 hours recovery time. Representative merged images are shown. (E) Confocal imaging of PMEF::HA-Dicer cells stained with p-DCR-1, 3F10, and γH2A.X antibodies following γ-irradiation with a total dose of 20 Gy and 0.5 hours recovery time. Quantitations indicate cells with shown phenotype in % and number of cell analysed (n).
(TIF)

S8 Fig Accumulation of nuclear phosphorylated HA-Dicer in PMEF::HA-Dicer cells upon γ-irradiation. (A) Immunoblot detecting phosphorylated HA-Dicer (p-DCR-1) in PMEF::HA-Dicer whole cell extracts in presence or absence of γ-irradiation (left) and immunoblots displaying reactivity of p-DCR-1 antibodies following incubation with whole cell extracts of wild type PMEFs, PMEF::HA-Dicer cells or Dicer^-/-^ knockout MEFs upon γ-irradiation (right); H3, histone 3, loading control; #, unspecific signal. (B and C) RGB profiles (B) and 3D reconstitution of super-resolution microscopy imaging (C) for phosphorylated HA-Dicer (p-DCR-1, green) or total HA-Dicer (3F10, red) in non-irradiated (Control) cells or cells irradiated with 10 Gy followed by 0.5–5 hour recovery time. Representative images are shown.
(TIF)
