# Supplementary figures and images for: Nuclear re-localization of Dicer in primary mouse embryonic fibroblast nuclei following DNA damage
Source: PLoS Genet. 2018 Feb 2;14(2):e1007151. doi: 10.1371/journal.pgen.1007151 (PMC5812656; doi:10.1371/journal.pgen.1007151)

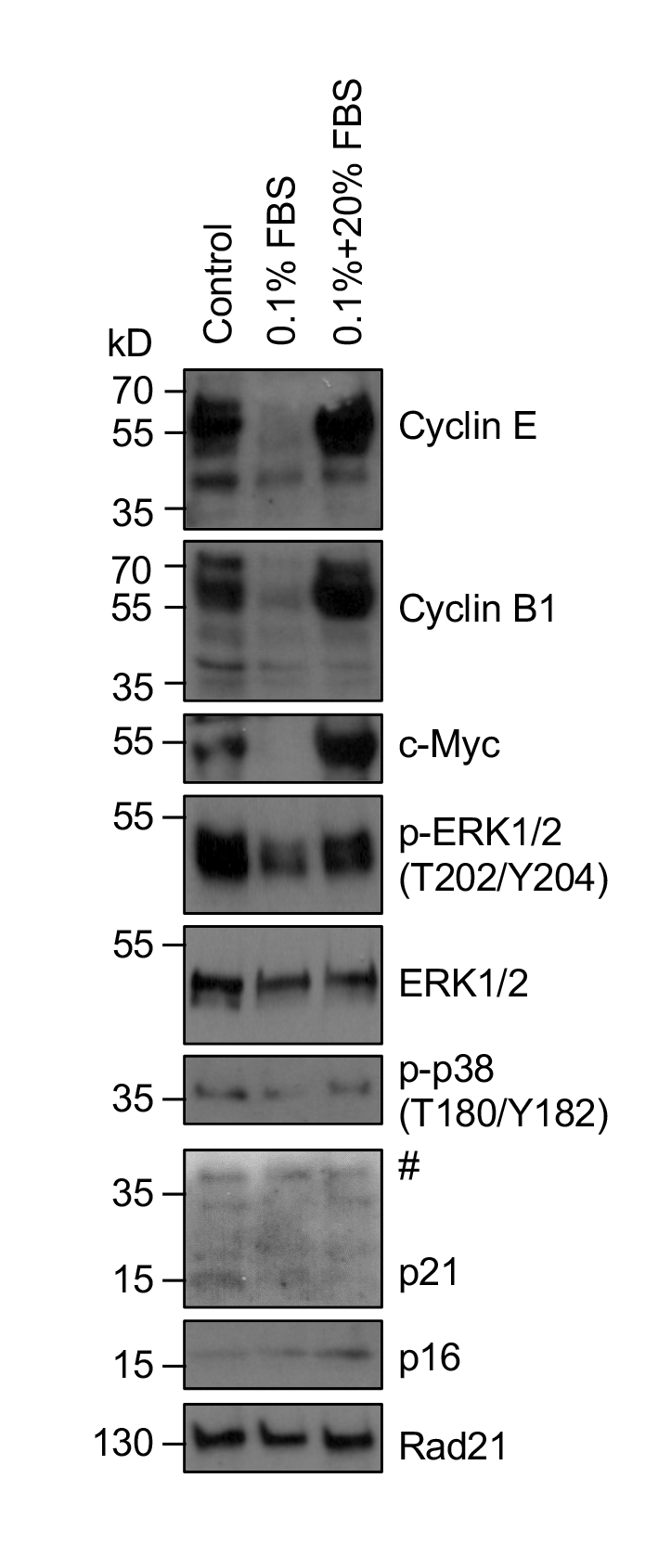

Supplement: S1 Fig — Immunoblots probing for cyclin E, cyclin B1, c-Myc, as well as total and phosphorylated ERK1/2 kinases, phosphorylated p38 kinase, or cell cycle inhibitors p21 and p16 in PMEF::HA-Dicer whole cells extracts following starvation (0.1% fetal bovine serum, FBS) or serum stimulation (0.1% FBS +20% FBS). Control, 10% FBS; Rad21, cohesin subunit, loading control; #, unspecific signal. (TIF) [file pgen.1007151.s001.tif]

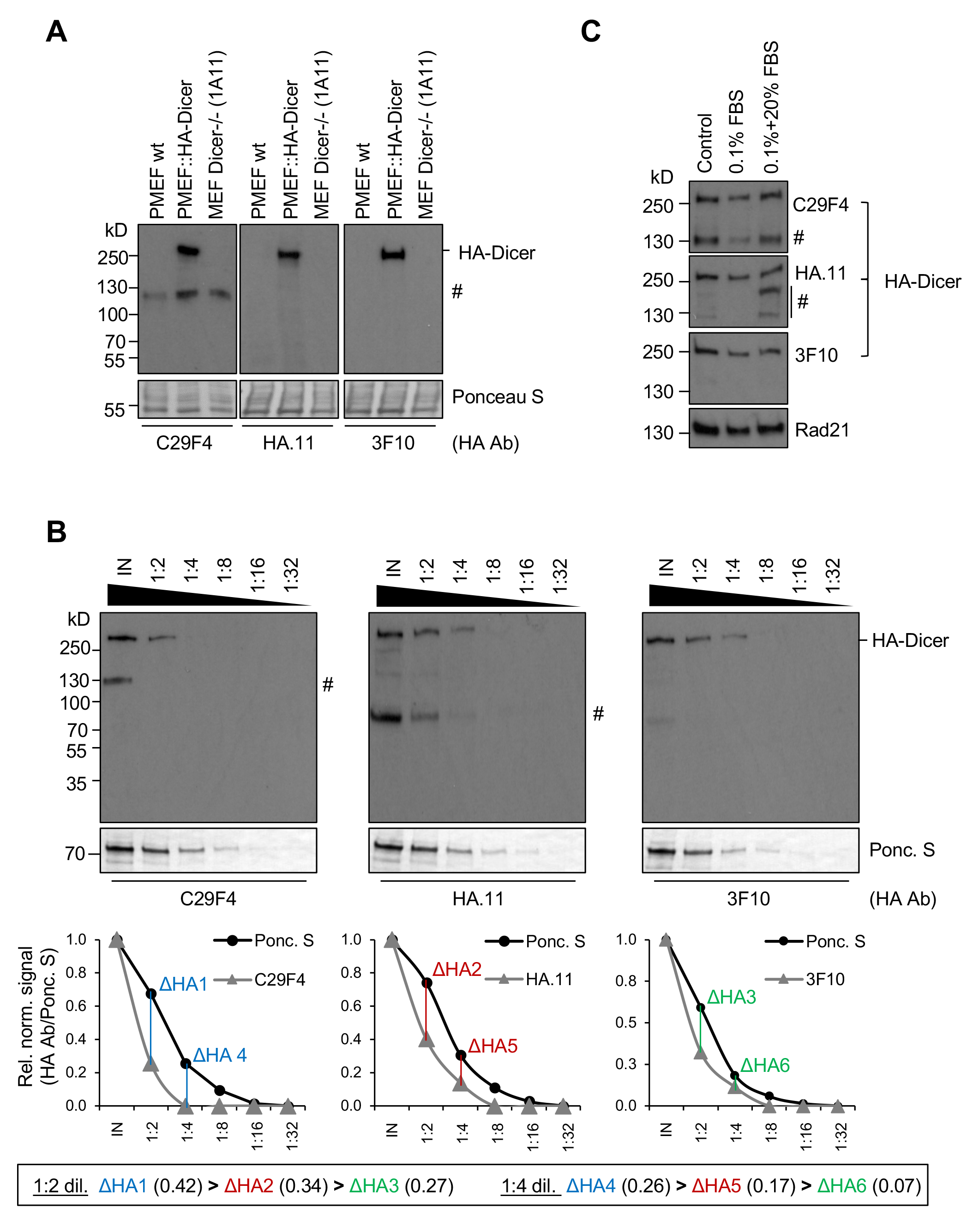

Supplement: S2 Fig — (A and B) Immunoblots displaying reactivity of HA antibodies C29F4, HA.11, 3F10 following incubation with (A) whole cell extracts of wild type PMEF, PMEF::HA-Dicer or Dicer-/- knockout MEF cells (clone 1A11) and with (B) serial dilution of PMEF::HA-Dicer whole cell extract. HA signals were quantified as arbitrary units relative to Ponceau S staining and normalised to input (IN, undiluted whole cell extract, 10% of lysate is loaded). Loss of reactivity of HA antibodies is defined as deltaHA (ΔHA = Rel. norm. Ponc. S signal—Rel. norm. HA Ab signal). Values at non-diluted input samples were set to 1. Ponceau S, loading control; #, aberrant signals; Ab, antibody. See also section “Materials and Methods” for details. (C) Immunoblots detecting total HA-Dicer (C29F4, HA.11, 3F10) in PMEF::HA-Dicer whole cells extracts following starvation (0.1% FBS) or serum stimulation (0.1% FBS +20% FBS). Control, 10% FBS; Rad21, cohesin subunit, loading control; #, aberrant signals. (TIF) [file pgen.1007151.s002.tif]

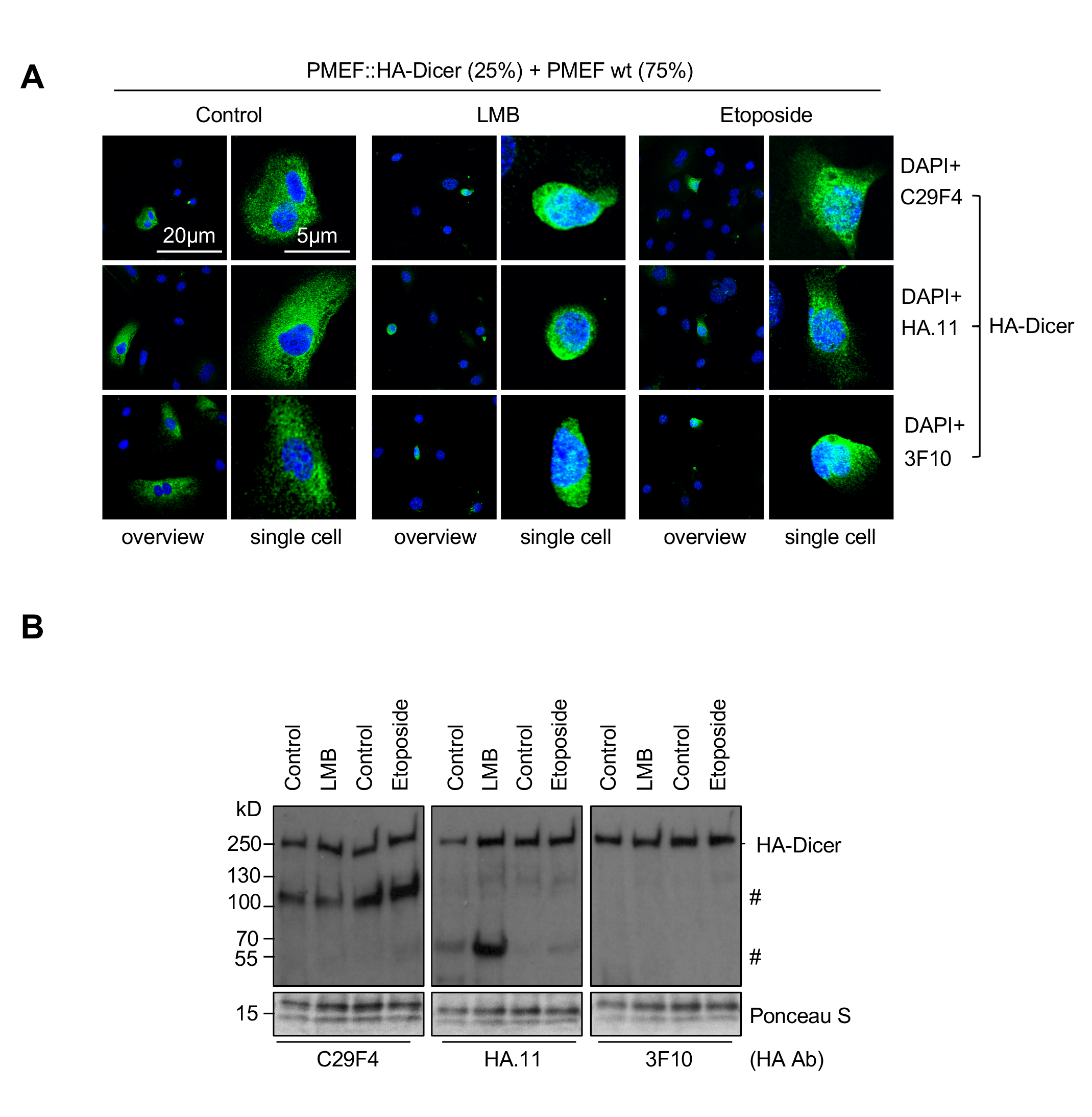

Supplement: S3 Fig — (A) Confocal imaging of wild type PMEF and PMEF::HA-Dicer co-cultures using HA antibodies C29F4, HA.11 and 3F10 in absence or presence of Leptomycin B (LMB) or Etoposide. Representative merged images are shown. (B) Immunoblots displaying reactivity of HA antibodies C29F4, HA.11 and 3F10 incubated with whole cell extracts of PMEF::HA-Dicer cells following treatment with Leptomycin B (LMB) or Etoposide. #, aberrant signals; Ab, antibody. (TIF) [file pgen.1007151.s003.tif]

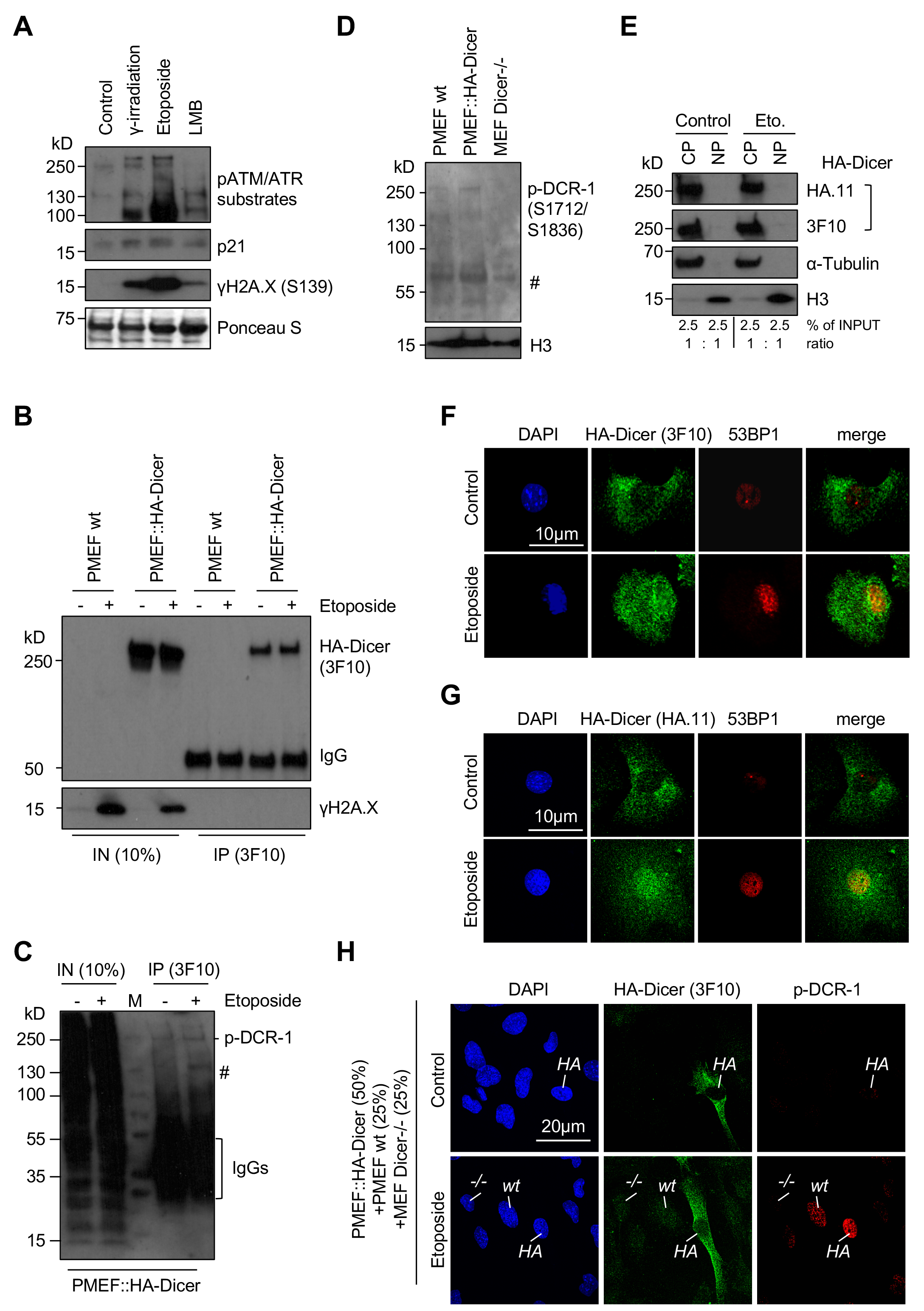

Supplement: S4 Fig — (A) Immunoblots detecting substrates of Ataxia-telangiectasia mutated (ATM) and ATM-related (ATR) kinase activity (pATM/ATR substrates mix antibody), p21 and phosphorylated histone variant H2A.X (γH2A.X, Ser139) levels in PMEF::HA-Dicer whole cell extracts following γ-irradiation (10 Gy, 2 hours recovery time) or incubation with Etoposide or Leptomycin B (LMB). (B) Immunoblots displaying reactivity of HA antibody 3F10 and γH2A.X levels after incubation with whole cell extracts (IN, input, 10% of lysate is loaded) of wild type PMEFs or PMEF::HA-Dicer cells or after immunoprecipitation (IP) using 3F10 antibody in absence or presence of Etoposide. IgG, immunoglobulin heavy chain, loading control. (C) Immunoblot detecting phosphorylated HA-Dicer using p-DCR-1 antibodies following IP with 3F10 antibody as described in (B); #, aberrant signal; M, molecular-weight size marker. (D) Immunoblots displaying reactivity of p-DCR-1 antibodies following incubation with whole cell extracts of wild type PMEFs, PMEF::HA-Dicer cells or Dicer-/- knockout MEFs after treatment with Etoposide. H3, histone 3, loading control; #, unspecific signal. (E) Immunoblots detecting total HA-Dicer (HA.11, 3F10) in subcellular fractions of PMEF::HA- Dicer cells in absence or presence of Etoposide. H3, histone H3; CP, cytoplasm; NP, nucleoplasm; CP and NP fractions are loaded in a 1:1 ratio. See also section “Materials and Methods” for details. (F and G) Confocal images showing PMEF::HA-Dicer cells stained for p53 binding protein 1 (53BP1) and total HA-Dicer using HA antibody 3F10 (F) or HA.11 (G) in absence or presence of Etoposide. Representative images are shown. (H) Confocal imaging of wild type PMEF (wt), PMEF::HA-Dicer (HA) and Dicer-/- MEF (-/-) co-cultures using 3F10 and p-DCR-1 antibodies in absence or presence of Etoposide. Representative merged images are shown. (TIF) [file pgen.1007151.s004.tif]

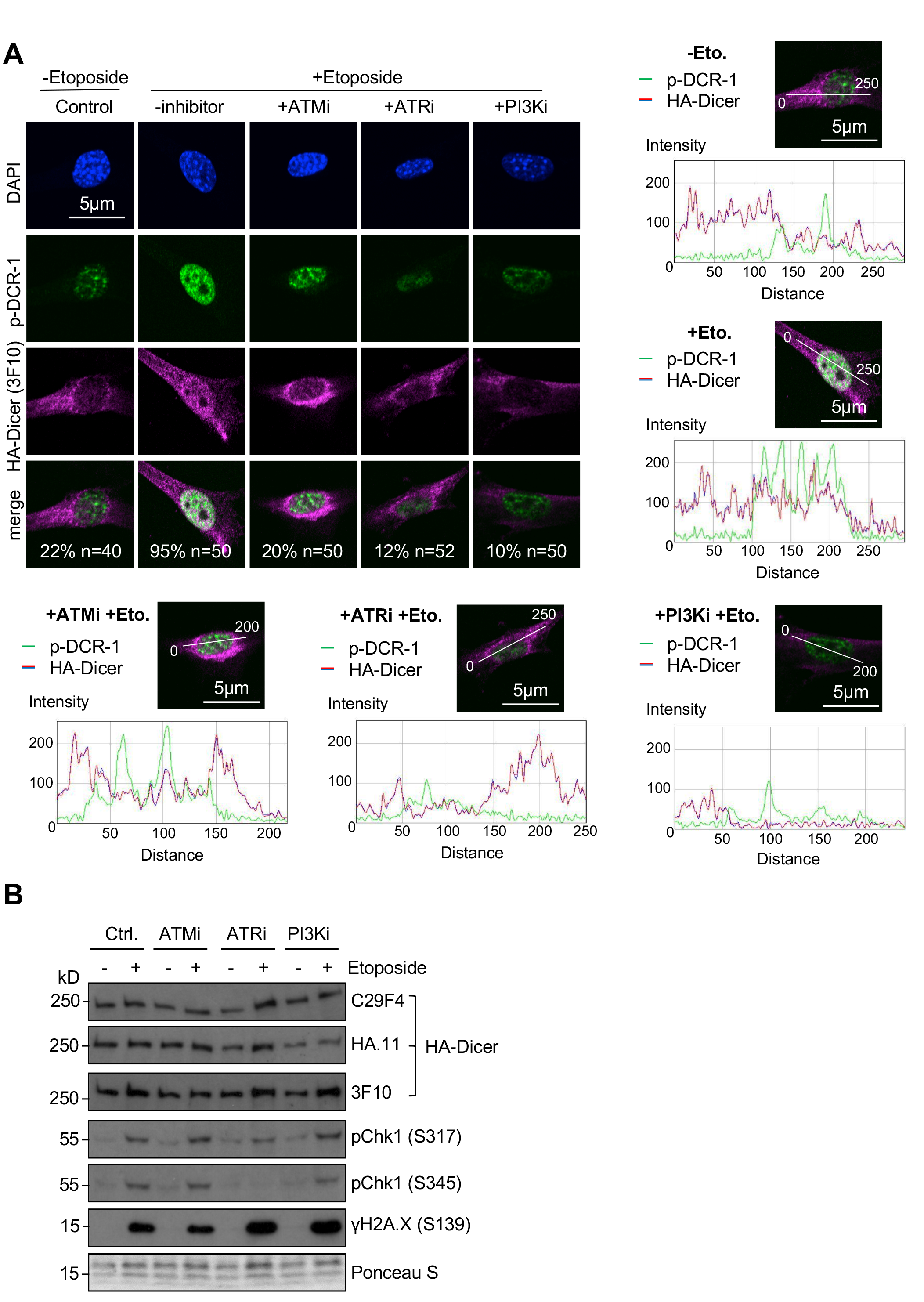

Supplement: S5 Fig — (A) Confocal imaging of phosphorylated HA-Dicer (p-DCR-1) and total HA-Dicer (3F10) in PMEF::HA-Dicer cells in presence or absence of Etoposide or after preincubation with Phosphatidylinositol-3-kinase (PI3K) inhibitors. Quantitation indicates cells with shown phenotype in % and number of cell analysed (n) (top panel). RGB profiles of p-DCR-1 (green) and HA-Dicer (red and blue) signals in representative cells. (B) Immunoblots detecting total HA-Dicer (3F10, HA.11, C29F4) as well as levels of phosphorylated checkpoint kinase 1 (pChk1, Ser317/Ser345) and γH2A.X in PMEF::HA-Dicer cells in presence or absence of Etoposide or after preincubation with Phosphatidylinositol-3-kinase (PI3K) inhibitors. Ponceau S, loading control. (TIF) [file pgen.1007151.s005.tif]

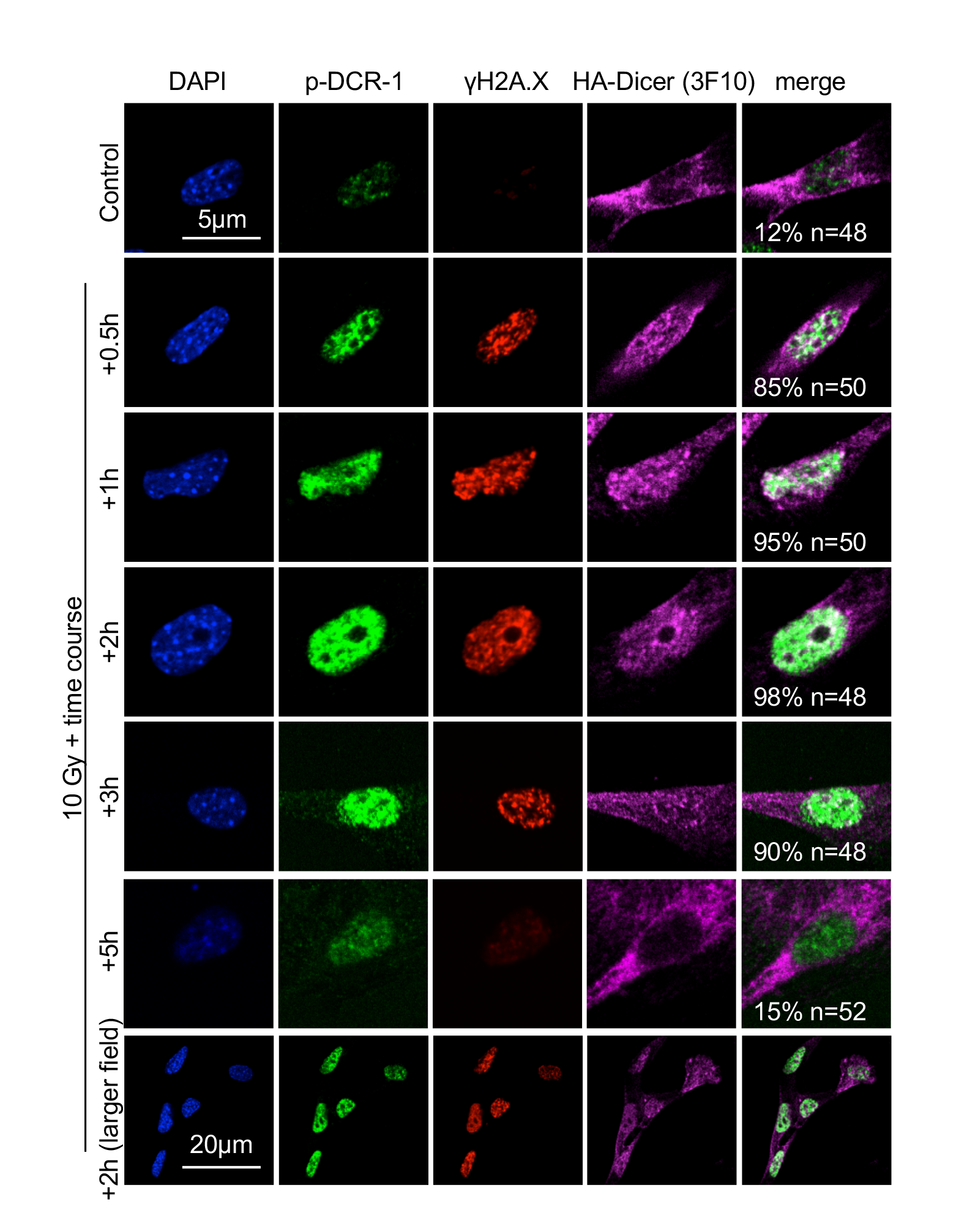

Supplement: S6 Fig — Time course confocal imaging of PMEF::HA-Dicer cells stained for phosphorylated Dicer (p-DCR-1), γH2A.X and total HA-Dicer (3F10) following γ-irradiation with a total dose of 10 Gy and recovery for various hours. Quantitation indicates cells with shown phenotype in % and number of cell analysed (n). (TIF) [file pgen.1007151.s006.tif]

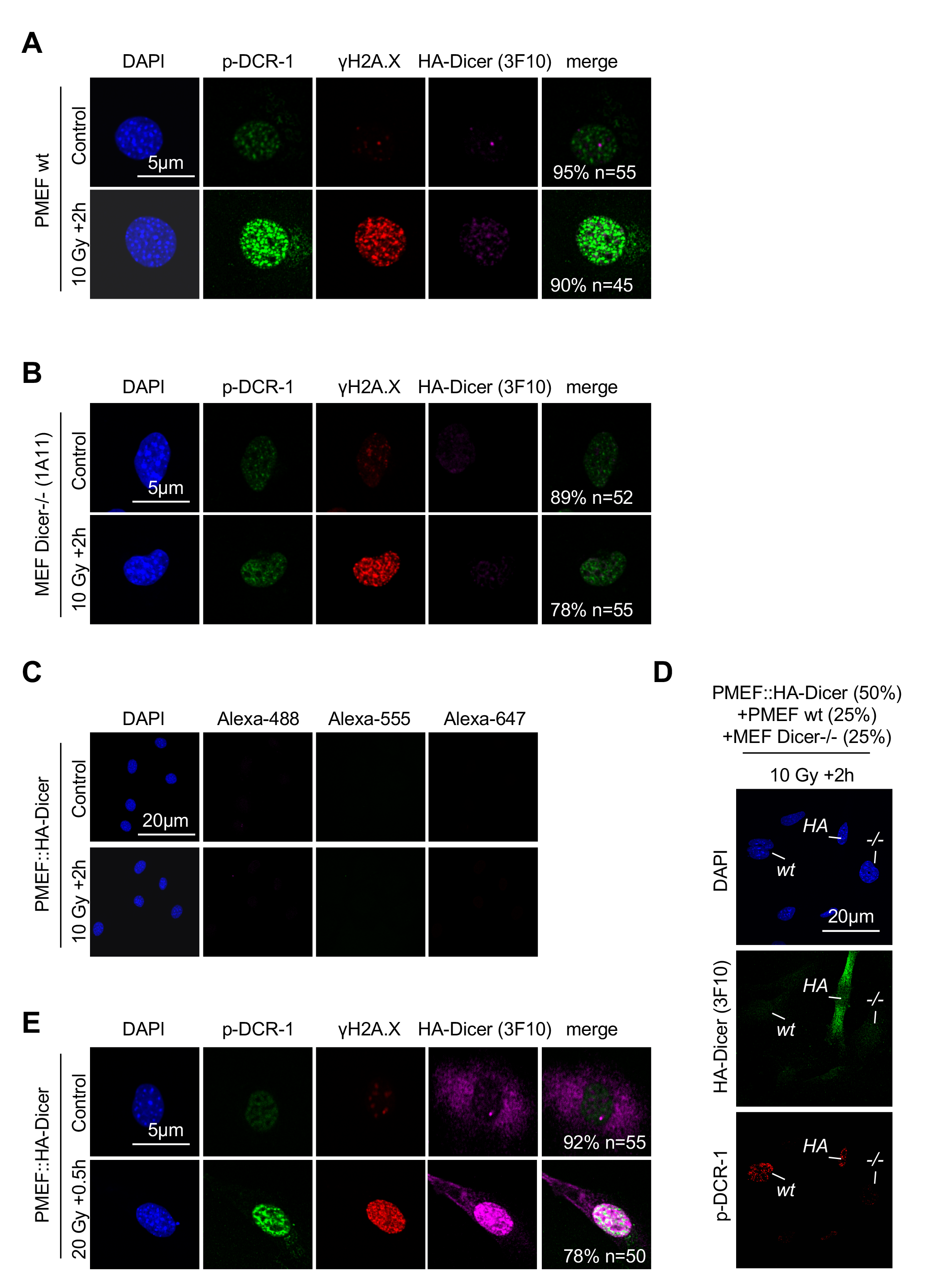

Supplement: S7 Fig — (A) Confocal imaging of wild type PMEF cells stained for phosphorylated Dicer (p-DCR-1), γH2A.X and total HA- Dicer (3F10) following γ-irradiation with a total dose of 10 Gy and 2 hours recovery time. (B) as in (A), but using Dicer-/- MEFs (clone 1A11). (C) Confocal imaging of PMEF::HA- Dicer cells incubated with Alexa-Flour conjugated secondary antibodies. (D) Confocal imaging of wild type PMEF (wt), PMEF::HA-Dicer (HA) and Dicer-/- MEF (-/-) co-cultures using 3F10 and p-DCR-1 antibodies following γ-irradiation with a total dose of 20 Gy and 2 hours recovery time. Representative merged images are shown. (E) Confocal imaging of PMEF::HA-Dicer cells stained with p-DCR-1, 3F10, and γH2A.X antibodies following γ-irradiation with a total dose of 20 Gy and 0.5 hours recovery time. Quantitations indicate cells with shown phenotype in % and number of cell analysed (n). (TIF) [file pgen.1007151.s007.tif]

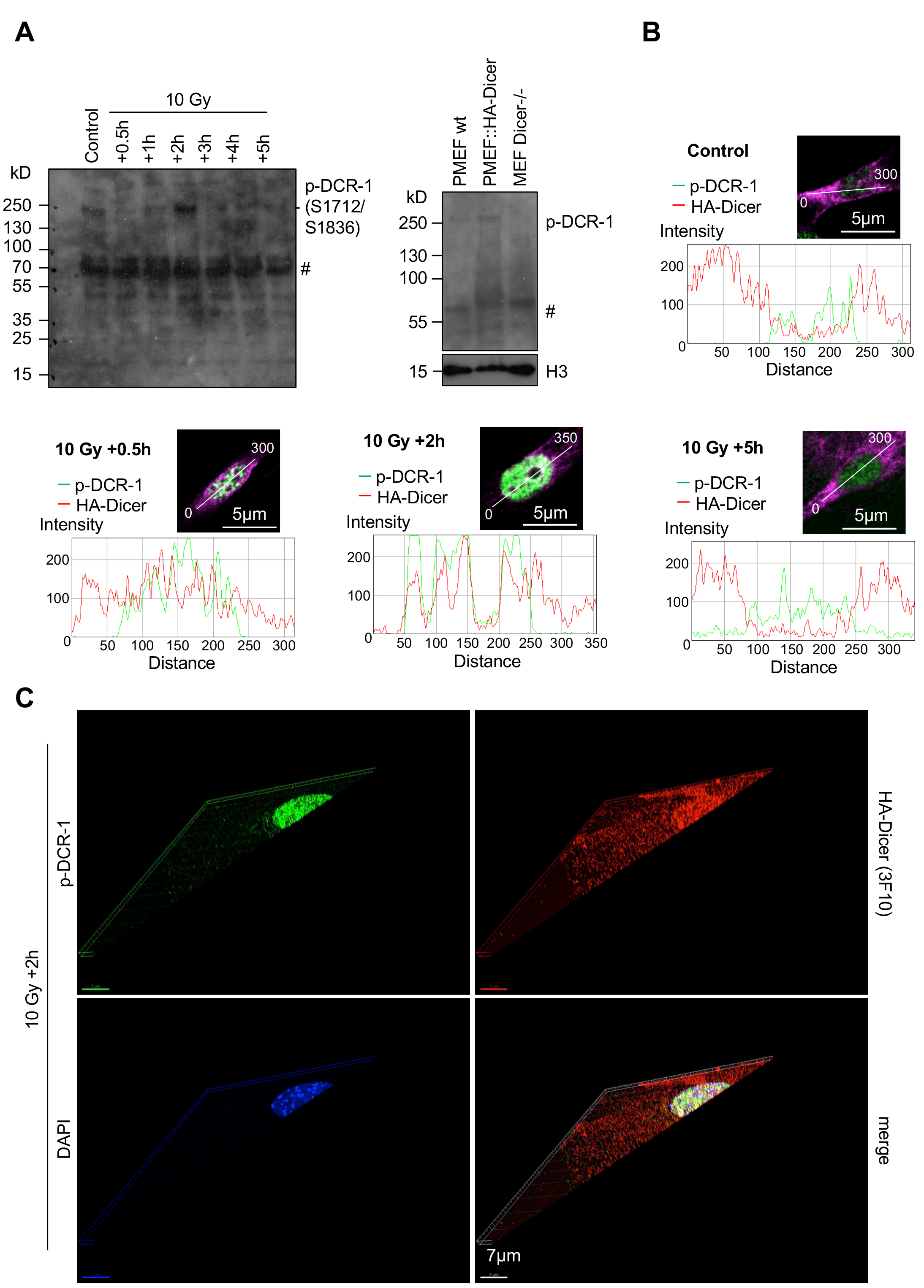

Supplement: S8 Fig — (A) Immunoblot detecting phosphorylated HA-Dicer (p-DCR-1) in PMEF::HA-Dicer whole cell extracts in presence or absence of γ-irradiation (left) and immunoblots displaying reactivity of p-DCR-1 antibodies following incubation with whole cell extracts of wild type PMEFs, PMEF::HA-Dicer cells or Dicer-/- knockout MEFs upon γ-irradiation (right); H3, histone 3, loading control; #, unspecific signal. (B and C) RGB profiles (B) and 3D reconstitution of super-resolution microscopy imaging (C) for phosphorylated HA-Dicer (p-DCR-1, green) or total HA-Dicer (3F10, red) in non-irradiated (Control) cells or cells irradiated with 10 Gy followed by 0.5–5 hour recovery time. Representative images are shown. (TIF) [file pgen.1007151.s008.tif]
